# The Biology of Synovial Sarcoma: State-of-the-Art and Future Perspectives

**DOI:** 10.1007/s11864-021-00914-4

**Published:** 2021-10-23

**Authors:** Michele Fiore, Andrea Sambri, Paolo Spinnato, Riccardo Zucchini, Claudio Giannini, Emilia Caldari, Maria Giulia Pirini, Massimiliano De Paolis

**Affiliations:** 1 Alma Mater Studiorum – University of Bologna, Bologna, Italy; 2 IRCCS Azienda Ospedaliero Universitaria di Bologna, via Massarenti 9, 40138 Bologna, Italy; 3 IRCCS Istituto Ortopedico Rizzoli, Bologna, Italy

**Keywords:** Synovial sarcoma diagnosis, Synovial sarcoma genomics, Sarcoma epigenomics, Synovial sarcoma treatment

## Abstract

New molecular insights are being achieved in synovial sarcoma (SS) that can provide new potential diagnostic and prognostic markers as well as therapeutic targets. In particular, the advancement of research on epigenomics and gene regulation is promising. The concrete hypothesis that the pathogenesis of SS might mainly depend on the disruption of the balance of the complex interaction between epigenomic regulatory complexes and the consequences on gene expression opens interesting new perspectives. The standard of care for primary SS is wide surgical resection combined with radiation in selected cases. The role of chemotherapy is still under refinement and can be considered in patients at high risk of metastasis or in those with advanced disease. Cytotoxic chemotherapy (anthracyclines, ifosfamide, trabectedin, and pazopanib) is the treatment of choice, despite several possible side effects. Many possible drug-able targets have been identified. However, the impact of these strategies in improving SS outcome is still limited, thus making current and future research strongly needed to improve the survival of patients with SS.

## Introduction

Synovial sarcoma (SS) is a malignant mesenchymal neoplasm [1, 2]. Multipotent mesenchymal stem cells have been considered as putative originators for several years, but SS origins are still unknown [3–7].

Synovial sarcoma accounts for 5 to 10% of all soft tissue sarcomas (STSs), and it predominantly occurs in older children and young adults [2, 8–10]. In the pediatric population, SS is the most common non-rhabdomyosarcoma STS [11–13]. It is almost ubiquitarious, but its intra-articular occurrence is very uncommon [14, 15]. Synovial sarcoma can arise anywhere in the soft tissues, generally as a progressively expanding mass. The most common clinical presentation is a slow-growing lump in the soft tissues of the lower limb (46.1% in the National Cancer Institute’s Surveillance—NEER database [16•]), especially around the knee and the ankle. The head and neck region, abdominal wall, retroperitoneum, mediastinum, pleura, lungs, and other organs are less common locations.

Various symptoms may be related to different sites (such as difficulty in swallowing and breathing, or alteration of voice in the head and neck SS), although a painless swelling is the most frequent appearance. Pain may be related to the involvement of nerves or perilesional phlogosis in the advanced stages. Slow tumor growth and the apparent harmlessness of symptoms often lead to a delayed diagnosis.

Synovial sarcoma is characterized by local invasiveness and a propensity to metastasize. Nevertheless, at the time of diagnosis, less than 10% of cases present with metastases [17, 18]. However, there is a high incidence of late metastases [17], reported in up to 50–70% of cases [19]. Most metastasis develop in the lungs (80%), although bone (9.9%) and liver (4.5%) are the next most frequent locations [20]. While STS are known to primarily metastasize by hematogenous route to the lungs, lymph node metastasis is not uncommon in SS, with clinically detectable lymph node disease found in 1–27% of newly diagnosed patients [21–23]. Metastases were found to be more frequent in older patients [24].

## Imaging

Radiographs show no pathological findings in approximately 50% of cases of SS, but eccentric or peripheral calcifications may be identified in up to 30% of cases [12, 25, 26].

The ultrasound appearance of SS often reveals a focal, nodular, typically ovoid or slightly lobulated, solid but hypoechoic soft-tissue mass suggestive of an indolent process [27]. Prominent heterogeneity was reported in less than 20% of cases, with both homogeneous hypoechoic well-defined areas (reflecting cystic or necrotic change) and heterogeneous hyperechoic areas with irregular margins (corresponding to cellular areas of aggressive viable tumor, hemorrhage, calcification, or fibrosis) [27].

Computed tomography typically shows a heterogeneous, non-infiltrative mass with attenuation similar to or slightly lower than that of muscle [12, 28–31], often with punctate, peripheral calcifications [32, 33]. Calcifications may also be identified in metastasis, particularly in the lungs [33]. Heterogeneous post-contrast enhancement was reported in 89–100% of cases [29], helping to distinguish SS that initially appear as a cystic lesion or hematoma [32].

**Fig. 1:**
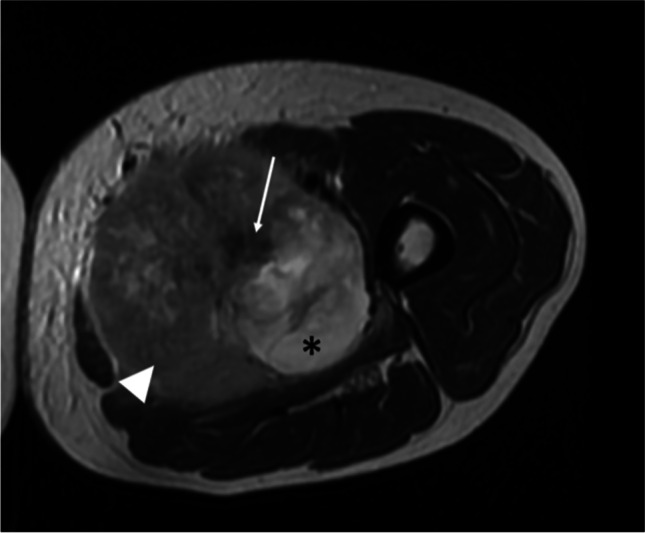
T2w axial MRI of the left thigh in a 54-year-old male, affected by synovial sarcoma with multiple lung metastasis at diagnosis, showed a large inhomogeneous mass with the so-called “triple sign”: fibrotic areas (low signal intensity — arrow), solid cellular elements (intermediate signal — arrowhead), and hemorrhage/necrosis areas (high signal intensity — asterisks)

**Fig. 2:**
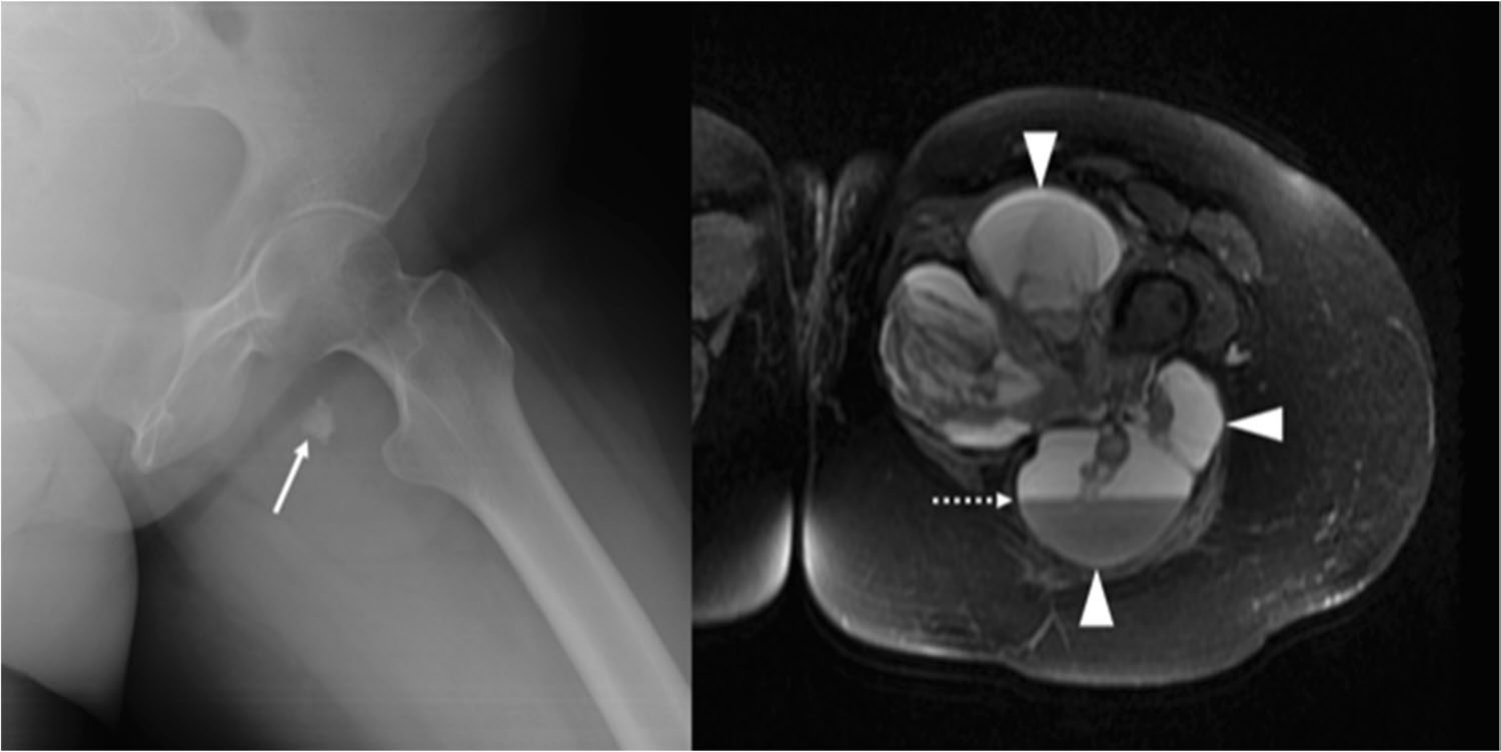
Conventional radiograph (left) and T2w axial fat-saturated MRI (right) of the left thigh in 32-year-old female with synovial sarcoma, showed calcific area (arrow), and the so-called “bowl of grapes” appearance with rounded cystic necrotic/hemorrhagic areas (arrowheads), containing a large fluid–fluid level (dotted arrow)

 Synovial sarcoma has a variety of magnetic resonance imaging (MRI) appearances, ranging from small, homogenous nodules to large heterogeneous masses encasing vessels and nerves. One study found that 33% of SS were less than 5 cm, and they had commonly benign imaging characteristics, with a predominantly homogeneous appearance on all MRI sequences [34]. On T1-weighted MRI images, SS typically appears as a heterogeneous multilobulated soft-tissue mass with signal intensity similar to or slightly higher than that of muscle [34–39]. Prominent heterogeneity (“triple sign”) is reported in up to 57% of cases [27, 29, 40]. It is represented by intermixed areas of low, intermediate, and high signal intensity on long repetition time images, as the result of the mixture of solid cellular elements, hemorrhage or necrosis, and calcified or fibrotic regions (Fig. 1) [40]. However, the “triple sign” lacks in specificity, as it is also seen in other STS, particularly in malignant fibrous histiocytoma [12]. Areas of hemorrhage, seen as fluid–fluid levels or foci of high signal intensity on T1- and T2-weighted MRI, are frequent. Fluid levels have been described in 10–25% of SS in several series [12]. This combination of features, particularly largely cystic areas or prominent hemorrhagic foci, often creates a “bowl of grapes” appearance (Fig. 2) [41]. Areas of calcification remain low-to-intermediate signal intensity on all MRI. MRI typically reveals conspicuous post-contrast enhancement in SS, usually heterogeneous, reflecting the intermixture of non-enhancing necrotic, cystic, or hemorrhagic regions and enhancing solid regions [39, 40].

Positron emission tomography (PET) of SS has been reported in few studies, but a marked increased tracer uptake was constantly described [42, 43].

Imaging may also play a role in prognostic assessment. In fact, several imaging features of SS have been found to be associated with poorer prognosis. In details, tumors larger than 5 cm, located more proximally (upper thigh, inguinal region, head and neck, and trunk), lack of calcification, intra-tumoral hemorrhage, and the presence of “triple sign” were found to be significantly associated with worse disease-free survival [29]. Early gadolinium enhancement on MRI (within 7 s after arterial enhancement) was also found to be associated with a more aggressive behavior [44]. FDG-PET may also provide prognostic elements because pre-treatment SUV greater than 4.4 was found to be associated with an increased risk of local recurrence and metastatic disease [45].

## Pathology

Macroscopically, SSs are multinodular masses, highly variable in size. Calcifications are common features, but they can be difficult to discern grossly. Occasionally, there are smooth-walled cysts containing mucoid fluid or blood. Hemorrhage and necrosis can be prominent in poorly differentiated SS, although less than in high-grade pleomorphic sarcomas [46].

Microscopically, three distinct subtypes are recognized: monophasic, biphasic, and poorly differentiated [47, 48]. Classification into subtypes is based upon somewhat subjective criteria, and there is a certain degree of overlap.

The monophasic type (the most common subtype) is composed of hypercellular arrays of small spindle cells with uniform, ovoid, vesicular nuclei with dispersed chromatin, inconspicuous nuclei, and very scarce amphophilic cytoplasm [2]. There is scarce intervening stroma, and the cells appear tightly packed. The stroma of SS can range from collagenous/hyalinized, rarely with amianthoid fibers [49], to extensively myxoid. An increase in hyalinized stromal collagen may be seen in neoplasms recurring after radiation therapy (RT) [50]. A hemangiopericytic vascular pattern, with sparse, dilated, thin-walled vessels, is seen in approximately 60% of cases [51]. Mast cells are relatively a characteristic feature of SS, but the presence of other inflammatory cells is atypical [50]. The monophasic epithelioid subtype, in which the histologic pattern is uniformly glandular, is rarely characterized [52–54], and it is histologically often indistinguishable from adenocarcinoma, requiring molecular confirmation for diagnosis.

Biphasic SSs consist of a mixture of both fibroblast-like spindle cells (similar in appearance to those of the monophasic spindle cell subtype) and epithelial cells (often forming gland-like structures). Although the proportions of the two components fluctuate, often, they are approximately the same. The epithelial cells have round or ovoid vesicular nuclei, moderate amounts of amphophilic cytoplasm, and distinct cell borders. The classical architecture of the epithelial element consists of relatively well-formed glands with lumina containing mucin that can form papillary structures with cores containing spindled tumor cells rather than connective tissue [50]. However, the epithelial components can also appear less well differentiated, forming solid nests.

The poorly differentiated subtype is highly cellular and usually comprises sheets of small, rounded cells, with hyperchromatic nuclei and amphophilic cytoplasm, with frequent mitotic activity and necrosis. A poorly differentiated component can be seen focally within SS [55], or it can account for the entire tumor, thus resembling other small round cell neoplasms. Two other types of poorly differentiated SS have been recognized: a large cell epithelioid variant, with polygonal cells with abundant cytoplasm, and a high-grade pleomorphic spindle cell variant [50, 56]. Poorly differentiated histology may occur more frequently in older adults [57].

Focal calcification, with or without ossification, is seen in approximately 30% of SS, more often in biphasic subtypes [50].

Synovial sarcoma has a differentiation score of 3, and it is therefore always a high-grade sarcoma (grade 2 or 3). Some authors proposed grading as an important prognostic factor [58].

## Genomic features

Synovial sarcomas harbor a highly specific, usually balanced and reciprocal translocation t(X;18)(p11.2;q11.2), in which the SS18 (formerly SYT) gene (18q11) fuses with SSX genes, leading to the generation of SS18-SSX fusion oncogenes [59–63]. Nine SSX genes (SSX1-9) have been described which are highly homologous [64].

SS18-SSX can be detected in more than 95% of SS [65], for which it is specific and has been seen in all morphologic subtypes. Approximately two-thirds of SS harbor SS18- SSX1 gene fusions and one-third SS18-SSX2 [47, 64, 66, 67].

The specific gene fusion has been shown to correlate with tumor histology. Almost all biphasic SSs have been shown to harbor SS18-SSX1 fusions [66–68], and almost all of the SS18-SSX2 tumors show absence of glandular differentiation (monophasic histology) [66]. The rearrangement has been shown to be present in both (epithelial and spindle) cellular components of biphasic SS [69].

Recent data suggest that fusion type does not have prognostic value [47, 70], despite earlier studies suggesting that SS18-SSX1 produces more aggressive disease than SSX2 [47, 71–74].

Fluorescence in situ hybridization (FISH) using an SS18 break-apart probe is currently the most widely used approach to demonstrate the presumptive presence of one of the SS18-SSX fusions. However, other approaches including RT-PCR and, increasingly, massive parallel sequencing, are being more widely used [75]. Although highly specific, RT-PCR and SS18 break-apart FISH that are not perfect and have had reported sensitivities as low as 94% and 83%, respectively [76, 77]. The use of both techniques has been recommended in the ancillary diagnosis of SS, giving at least 96% sensitivity and 100% specificity [55]. However, the rare cases of neoplasms morphologically and immunohistochemically typical of SS but without SS18-SSX fusions could represent tumors with unusual variant transcripts, which cannot be detected using routine molecular techniques [78].

Other than this translocation, SS tumors are mutationally quiet [79, 80]. Despite this, metastatic SSs are associated with increased tumor genomic instability [81, 82].

## Epigenomic

As mentioned above, the SS18-SSX fusion proteins are widely considered to be the main driver of SS pathogenesis [83, 84], as their expression is sufficient to induce SS tumors in mice [6, 72], and their silencing causes SS cells to revert to mesenchymal stem cell-like cells [5].

Recent efforts have focused on unraveling the mechanism behind the SS18-SSX-mediated epigenetic rewiring, focusing on the interplay between the SS fusion protein and the chromatin remodeling machinery, in particular with regard to the two key protein complex families of epigenetic modifiers: SWItch/Sucrose NonFermentable (SWI/SNF) and Polycomb Repressive Complexes (PRC) [79, 85, 86]. While PRC leads to chromatin compaction and gene repression, SWI/SNF complexes facilitate transcription by remodeling nucleosomes, thereby promoting gene activation by permitting increased access of transcription factors to their binding sites [87].

The SWI/SNF or BRG1/BRM-associated factor (BAF) complexes are members of a family of Trithorax-group proteins (TrxG) [88, 89]. Only one of the three mammalian SWI/SNF complexes, the canonical BAF (cBAF) complex, contains SS18 and has been shown to interact with the SS fusion proteins. The SS18-SSX fusion proteins have been shown to competitively replace the wild-type SS18 in the cBAF complexes [90, 91], thus resulting in ejection of SWI/SNF-related matrix-associated actin-dependent regulator of chromatin subfamily B member 1 (SMARCB1) and its subsequent proteasome-mediated degradation [92••]. These oncogenic BAF complexes are subsequently retargeted to PRC-repressed domains and have been shown to activate them [91], recruiting RNA Polymerase II to initiate transcription [93].

Another current theory is that the SS18-SSX oncoprotein mediates its transcriptional silencing via interaction with PRC1 and PRC2, since studies have shown SS18-SSX to co-localize with the complexes [94, 95]. The canonical PRC1 consists of two core subunits: RING1A/B and PCGF16. The PCGF components are important for maintaining the protein–protein interactions that initiate chromatin silencing [96, 97] and the knockdown of PCGF4 or either of the RING proteins, leading to a global reduction in PRC1 activity [98]. Concurrent with this, Barco et al. also found that the presence of SS18-SSX2 is associated with a downregulation of PCGF4 and subsequently with a decreased PRC1 activity [99]. There are also several heterogeneous non-canonical PRC1 complexes [86, 100]. In another proposed model, SS18-SSX utilizes lysine-specific demethylase 2B (KDM2B) as part of one of these non-canonical PRC1 (PRC1.1) to target cBAF to unmethylated CpG islands, generating a BAF-mediated PRC2 antagonism and aberrant gene activation at these sites [101]. PRC2 executes its chromatin silencing functions via its catalytic subunit Enhancer of Zeste 2 (EZH2), a histone methyltransferase [102]. SS18-SSX can serve a bridging function connecting activating transcription factor 2 (ATF2) to the PRC2 member transducin-like enhancer protein 1 (TLE1) and in doing so represses the expression of important tumor suppressor genes, including cyclin-dependent kinase inhibitor 2A (CDKN2A) and early growth response protein 1 (EGR1) [103–105].

There is currently no definitive theory for the pathogenesis of SS; however, the previous hypothesis shows that it could principally depend on the disruption of the balance of the complex interplay between the TrxG and PcG complexes. Therefore, a better understanding of the effects and consequences of the expression of SS18-SSX fusion proteins on the epigenomic regulators is needed [91, 106].

## Expression profile

Gene expression studies have also shown several differences between SS18-SSX1 and SS18-SSX2 fusion types, suggesting that these may lead to different downstream events [107].

Studies on the direct and indirect interactions of SS18-SSX oncoproteins suggest that they particularly affect cell growth and proliferation and have highlighted cyclin D1, Wnt/β catenin pathway components (LEF1, TCF7, ZIC2, WNT5A, AXIN2, and FZD10), TP53 pathway components, EGR1, insulin-like growth factor 2 together with its receptor IGF-1R, and chromatin remodeling mechanisms, as the most important targets of these oncoproteins contributing to sarcomagenesis [108].

However, an independent role might be played by the above-mentioned TLE1 gene (9q21.32), a member of the TLE family of genes that encode Groucho-like transcriptional corepressors. In fact, TLE1 is one of the most frequently overexpressed genes in SS [107, 109–111]. It binds other basic helix-loop-helix proteins to repress target genes [112–114] thus inhibiting the Wnt/βcatenin signaling and other cell fate determination signals and have an established role in repressing differentiation [115, 116].

Other genes and pathways that exhibit perturbations in SS include Hedgehog (SMO, PTCH1), NY-ESO-1 (CTAG1A), and Notch (JAG1, JAG2, and HES1) and RTKs (FGF2, FGF3, EGFR, PDGFR, and IGFBP3) [111, 117, 118]. Moreover, the propensity for epithelial differentiation has been associated to the derepression of the transcription of E-cadherin [65]. Nevertheless, 21 different microRNAs (including let-7e, miR-99b, and miR-125a-3p) were found significantly upregulated in SS, suggesting that also these molecules have a potential oncogenic role [119].

Because these pathways and genes are not consistently affected in all cases, efforts have been made to identify a genetic signature that predicts survival or tumor progression [80]. For example, a downregulation of genes associated with neuronal and skeletal development and cell adhesion, as well as the upregulation of genes on the 8q21.11 locus, were identified in poorly differentiated SS [111]. However, further characterization of expression profiles is needed to identify possible prognostic factors and potential therapeutic targets.

## Immunohistochemistry

A range of immunohistochemical (IHC) markers have been proposed to support the diagnosis of SS, most notably TLE1 [120]. However, to date, no single IHC marker or combination of markers can definitively confirm or exclude the diagnosis of SS [75]. Thus, despite FISH and molecular testing being expensive, not widely available, and time consuming in comparison to IHC, these approaches still represent the “gold standard” in SS diagnosis.

TLE1, due to its upregulation in SS, was identified from gene expression studies as a useful biomarker for distinguishing SS from other STSs [121]. TLE1 shows strong and diffuse nuclear staining in SS [116], with positive nuclear expression observed in more than 90% of cases [122, 123]. A recent systematic review examining the role of TLE1 as a diagnostic biomarker for SS found that the mean sensitivity and specificity of TLE1 in detecting SS were 94% (95% CI 91–97%) and 81% (95% CI 72–91%), respectively. The mean positive predictive value of TLE1 was 75% (95% CI 62–87%), whereas the negative predictive value was 96% (95% CI 93–98%) [118]. However, TLE1 expression has also been reported in up to one-third of non-SS [123], including potential mimics in differential diagnosis such as 17–20% of solitary fibrous tumors, 13–30% of malignant peripheral nerve sheath tumors, and 69% of malignant mesotheliomas [124] and, less commonly, 7% of carcinomas [120, 122, 125–127]. Nuclear TLE1 expression is also observed in non-neoplastic tissues, with variable expression in basal keratinocytes, adipocytes, perineurial cells, endothelial cells, and mesothelial cells [122]. Therefore, particularly when its expression is moderate or strong, TLE1 is helpful in distinguishing SS from its histologic mimics; however, it should be used only in the context of a panel of antibodies (including keratins, EMA, CD34, and bcl-2) [120, 122].

NY-ESO-1, a cancer testis antigen, is also strongly and diffusely expressed in most SS (as in 76% of tumors) but rarely in other mesenchymal lesions and may be useful in distinguishing SS from other spindle cell neoplasms [128, 129].

Brachyury transcription factor and CD34 are consistently negative in SS [130], while SMARCB1/INI1 protein expression was found to be reduced in 69% of cases, although no case with complete loss of expression was recognized [131, 132].

The diagnostic value of other markers has been limited by their lack of sensitivity and/or specificity. More than 90% of SS, including all histologic subtypes, show focal expression of epithelial markers cytokeratins and epithelial membrane antigen (EMA), with a characteristic patchy pattern in the spindle cell component and a more uniform staining in the epithelial component [133–135]. Cytokeratin subtypes CK7 and CK19 appear essentially restricted to SS and are helpful in their diagnosis [136–138]. As a significant number of SS are keratin positive but EMA negative or vice versa [133, 134], both markers should be used in a complementary manner. Other immunomarkers with some utility include carcinoembryonic antigen (CEA), vimentin, calponin, Bcl2, CD99, and S100 protein [80, 116, 120–123, 128, 129, 131, 132, 135, 139–141].

Two new rabbit monoclonal antibodies have recently been developed and proposed to be highly sensitive and specific for the diagnosis of SS [142]: E9X9V (cat no 72364, Cell Signaling Technology, Danvers, MA USA) designed to recognize the SS18-SSX fusion proteins without cross reacting with wild-type SS18 or SSX proteins and E5A2C, and E5A2C (cat no 23855, Cell Signaling Technology, Danvers MA USA) designed to recognize the C-terminal end of the SSX1, SSX2 and SSX4 proteins [142]. If validated, these results could lead to introduce these antibodies in clinical practice to support SS diagnosis.

## Prognosis

SS is generally considered a high-grade sarcoma, marked by a poor prognosis, with an overall survival (OS) rate of 87.3% at 1 year, 59.4% at 5 years, 50.8% at 10 years and 42.8% at 20 years follow-up, according to a recent large series [16•]. The difference between medium- to long-term survival reflects the fact that metastases in SS often occur very late, even beyond 10 years [17]. Patients with metastasis at diagnosis have a very poorer prognosis, with a 3-year survival rate of 27.2% [143].

Older patients, primary tumor located to the trunk, and large tumor size have been consistently reported to be associated with worse outcomes [24, 144, 145]. Tumor site can also affect prognosis, with a worse outcome for tumors arising from anatomic sites other than the extremities [41, 146–148].

Xiong et al. reported higher 5- and 10-year survival rates in the biphasic subtype (69% and 60%, respectively), followed by the monophasic subtype (59% and 49%, respectively) and lowest in the epithelioid subtype (32% and 26%, respectively) [149]. Bianchi et al. confirmed this observation, also reporting a worse survival in patients affected by FNLCC grade 3 SS than in those with grade 2 SS [58].

## Treatment

Standard treatment of primary, localized SS is represented by wide surgical resection of the tumor. However, there is conflicting evidence regarding the systemic benefits of adjuvant RT [150–153]. Some prospective, randomized controlled studies on STS show evidence that adjuvant RT improves local control but not OS [151, 154, 155]. Specifically to SS, Rhomberg et al. observed that SS might be resistant to RT [156]. However, Seo et al. observed that RT is more effective in a subgroup with characteristics of old age (age > 20 years), male patients, large tumors (> 5 cm), extremity locations, early stages, and biphasic subtypes [157]. These data can partially support routine implementation of RT in the multimodality treatment of patients with SS [151].

The combination of RT combined with chemotherapy (ChT) can be significantly toxic, including risk of treatment-related deaths. Therefore, when treating patients with SS, clinicians may choose to forego RT and/or combination ChT with RT in favor of ChT alone as the combination of modalities increases toxicity and may lead to ChT dose reduction.

### Cytotoxic chemotherapy

Cytotoxic ChT is often considered in both the neoadjuvant and adjuvant settings for patients with advanced SS [148]. Ferrari et al. reported 5-year, metastasis free survival rates of 60 and 40% for patients treated with and without ChT, respectively [158]. Edmonson et al. showed partial tumor regression in 5 of 12 patients with residual, recurrent, or metastatic tumors, with a median OS of 11 months [159].

Combined treatment with doxorubicin and ifosfamide represent front-line therapy for SS, with an expected response rate (RR) ranging between 25 and 60% [160–162]. For patients not amenable to anthracycline, single-agent high-dose ifosfamide is a valid alternative option, as it is in patients already pretreated with ifosfamide [163].

In some cases, ifosfamide monotherapy can be considered after this first-line combination treatment, in particular when there has been a reasonable interval between the end of first- and the start of second-line treatment. Single-center data on ifosfamide rechallenge in different STS subtypes showed the highest activity in SS [164]. In less fit patients, sequential doxorubicin and ifosfamide can be considered.

Recently, evofosfamide, a hypoxia-activated prodrug of bromo-isophosphoramide mustard, was evaluated within a randomized phase III trial in STS, which included patients with SS, who were randomly assigned to receive doxorubicin alone or doxorubicin plus evofosfamide. Evofosfamide showed an improvement in OS for the SS despite no evidence of survival benefit in the overall population [165].

An alternative treatment is the combination of gemcitabine and docetaxel, which may be considered in patients who cannot tolerate or are resistant to standard protocols. However, early studies suggested that gemcitabine, despite its effectiveness in STS, might not have much activity in SS [166, 167]. Similarly, in an early randomized study, patients receiving docetaxel exhibited no discernible responses [168, 169].

In second and later lines, trabectedin demonstrated antitumor effect in SS, with a 6-month progression-free survival (PFS) rate of 22% to 23% in two different retrospective studies and a 15% RR [170, 171]. The mechanism of action is still being elucidated; it may affect transcription factors and tumor microenvironment through neoplastic macrophage depletion [172, 173].

### Molecular targets

Tyrosine kinase inhibitors (TKIs) have some activity in SS, but pazopanib is the only one approved for treatment of STS. Pazopanib is an oral, multi-targeted tyrosine kinase inhibitor directed against the receptor tyrosine kinases (RTKs) vascular endothelial growth factor receptors (VEGFR) 1/2/3, platelet-derived growth factor receptors (PDGFR) α/β, and KIT, thereby blocking tumor growth and inhibiting angiogenesis. In the randomized phase III registration study, pazopanib was administered to 38 patients affected by SS and, compared with placebo, it improved the median PFS of 3 months (4.1 vs 1.0 month) [174–176]. Recent phase II and III studies suggest that pazopanib has activity in metastatic and refractory SS [177, 178]. Another TKI under investigation in STS is the multikinase VEGFR/PDGFR inhibitor regorafenib [179, 180]. Finally, there are anecdotal reports on the activity of sorafenib and sunitinib and of bevacizumab combined with cediranib [181–183]. Although pazopanib and regorafenib were reported to significantly improve PFS compared with placebo in advanced SS patients, these treatment strategies did not improve the OS [179, 184, 185]. Apatinib is an oral anti-angiogenesis TKI, a highly and selective inhibitor on VEGFR with promising efficacy in advanced SS patients, although the evidence level of this study seems preliminary [186••, 187, 188]. Other trials have been designed to inhibit specific targets in SS, in particular VEGF antibodies and the IGF-1R antibody cixutumumab [80, 189]. Olaratumab, a selective PDGFR monoclonal antibody, showed promising results in combined regimens [190]. In addition, PDGFR expression was recently reported in 84% of 44 SS tumor samples evaluated with immunostaining [191].

A new class of drugs able to inhibit EZH2 (the catalytic component of PRC2) is presently under investigation in tumors with BAF47/INI1 loss. The results of a phase II study of EZH2 inhibitor tazemetostat in the cohort of 33 patients with SS unluckily showed only a limited antitumor effect, with no objective responses and a 5-month median PFS [192].

Radiotherapy induces DNA double-strand breaks, stimulating DNA repair mechanisms, particularly those involving HDAC [193]. In preclinical studies, HDAC inhibitors induced differentiation, apoptosis, and growth arrest of SS cells while increasing tumor cell sensitivity to RT and ChT [103, 194, 195]. A phase II trial (NCT00112463) to study the efficacy of an HDAC inhibitor (romidepsin) in SS has recently closed to accrual, and results of the trial are pending [80].

Preclinical studies suggested several other actionable targets in SS, among which are the WNT-b-catenin and the protein kinase B (AKT)-mammalian target of rapamycin (mTOR) pathways, anaplastic lymphoma kinase (ALK), MET, and the cyclin D1-CDK4/6-Rb axis [117, 191, 196–200]. Moreover, various epigenomic regulators such as BCOR (a PRC1.1 component) [100] as well as SKP2 (an E3 ubiquitin ligase) were found to be overexpressed in undifferentiated SS, thus being a potential targetable gene [201].

However, despite the promising preclinical studies, the translation of these results to improved clinical outcomes remains challenging, and the benefit achieved from the introduction of new agents for management of advanced SS has been limited over the last decade.

### Immunotherapy

Programmed death-1 protein (PD-1) is normally expressed on the surface of activated T-cells and suppresses unwanted or excessive immune responses, including autoimmune reactions. Its ligand PD-L1 can be expressed by various cells, including macrophages and tumor cells. The PD-1/PD-L1 interaction is a major pathway used by tumors to suppress immune control. Several studies have assessed the expression of PD-L1 in sarcomas [202]. However, a recent study by Pollack et al. [203] demonstrated that among STS, SS has the lowest expression of PD-1/PD-L1 and the lowest T-cell infiltration [204]. This explains different trials with pembrolizumab, ipilimumab, and nivolumab demonstrated no activity of cytotoxic T-lymphocyte antigen 4 (CTLA4) or PD-1 inhibition for the treatment of SS [205, 206]. Nonetheless, Jerby-Arnon et al. recently reported a novel “core oncogenic program” driven by SS18-SSX, with implications for treatment strategies based on epigenetics, cell-cycle control, and immune augmentation [204]. Therefore, further studies might examine whether HDAC and CDK4/6 inhibitors could induce T-cell priming and recruitment due to cell damage and test potential synergies with different forms of cancer immunotherapies, such as immune checkpoint blockade, adoptive T-cell therapies, or cancer vaccines. Several clinical trials evaluating the efficacy of these new therapeutic approaches are currently ongoing. Thus far, it has been reported that trials with more targeted immunotherapies against tumor-specific antigens have shown greater promise in SS, in particular vaccines that trigger priming of NY-ESO-1-specific T-cell response [207], as well as therapies based on autologous T-cells transduced with a T-cell receptor directed against NY-ESO-1 [208].

### Metabolic therapy

Arginino-succinate synthetase 1 (ASS1) is the rate-limited enzyme in the urea cycle responsible for the formation of arginine-succinate from citrulline and aspartate. When ASS1 is not expressed, cells are reliant on extracellular sources of the aminoacid arginine. Loss of expression of ASS1 due to methylation has been demonstrated to be the most common defect among STS, including SS [209]. This loss makes SS an attractive cancer for treatment with arginine starvation with agents such as pegylated arginine deiminase [210]. Arginine starvation alters SS metabolism and glutathione levels, making it more sensitive to treatment with ChT [210]. This metabolic defect is under development as the basis for a multiagent biomarker-driven metabolic therapy for SS.

## Conclusions

Substantial advances in the understanding of the natural history and pathogenesis of SS have been made. However, the prognosis is still scarce.

The standard of care for primary SS is wide surgical resection combined with RT in selected cases. The role of ChT is still under refinement and can be considered in patients at high risk of metastasis or in those with advanced disease. Cytotoxic ChT (anthracyclines, ifosfamide, trabectedin, and pazopanib) are the treatments of choice, despite several possible side effects. Many possible drug-able targets have been identified. However, the impact of these strategies in improving SS outcome is still limited, thus making current and future research strongly needed to improve the survival of patients with SS.

## Data Availability

Data were obtained from Literature.
